# Spatial Variability of Rare Earth Elements in Groundwater in the Vicinity of a Coal-Fired Power Plant and Associated Health Risk

**DOI:** 10.3390/toxics12010062

**Published:** 2024-01-12

**Authors:** Jelena Vesković, Milica Lučić, Mirjana Ristić, Aleksandra Perić-Grujić, Antonije Onjia

**Affiliations:** 1 Faculty of Technology and Metallurgy, University of Belgrade, Karnegijeva 4, 11120 Belgrade, Serbia; 2 Innovation Center of the Faculty of Technology and Metallurgy, Karnegijeva 4, 11120 Belgrade, Serbia

**Keywords:** hazard index, ILCR, TPP, GIS, correlation analysis, anomalies, cancer risk, REEs, REY, heavy metal(loid)s

## Abstract

This study investigated the occurrence and distribution of rare earth elements (REEs), including 14 lanthanoids, scandium (Sc), and yttrium (Y), in groundwater around a large coal-fired thermal power plant (TPP). The ICP-MS technique was used to analyze 16 REEs in groundwater samples collected from monitoring wells. REE concentrations ranged from 59.9 to 758 ng/L, with an average of 290 ng/L. The most abundant was Sc, followed by La, accounting for 54.2% and 21.4% of the total REE concentration, respectively. Geospatial analysis revealed the REE enrichment at several hotspots near the TPP. The highest REE concentrations were observed near the TPP and ash landfill, decreasing with the distance from the plant and the landfill. REE fractionation ratios and anomalies suggested the Light REE dominance, comprising over 78% of the total REEs. Correlation and principal component analyses indicated similar behavior and sources for most REEs. Health risk assessment found hazard indices (HI) of 1.36 × 10^−3^ and 1.98 × 10^−3^ for adults and children, respectively, which are far below the permissible limit (HI = 1). Likewise, incremental lifetime cancer risks (ILCR) were all below 1 × 10^−6^. Nevertheless, ongoing ash disposal and potential accumulation in the environment could elevate the REE exposure over time.

## 1. Introduction

The group of elements known as rare earth elements (REEs) is composed of lanthanoid elements, which range from lanthanum (La) to lutetium (Lu), along with scandium (Sc) and yttrium (Y) [1,2]. These elements are typically classified based on their atomic radii and masses. This classification results in three groups: the light REEs (LREEs), which comprise lanthanoids from lanthanum (La) to neodymium (Eu), including scandium; the middle REEs (MREEs), which include lanthanoids from samarium (Sm) to dysprosium (Dy); and the heavy REEs (HREEs), which comprise lanthanoids from holmium (Ho) to lutetium (Lu), including yttrium [3,4].

Rare earth elements are extensively utilized in numerous sectors, such as industry, agriculture, and technology, due to their distinctive physical and chemical characteristics. They play a role in producing and functioning a wide array of items, including magnets, cell phones, fertilizers, batteries, pigments, electric cars, Al-Mg alloys, etc. [5,6,7]. As a result, the demand for REEs has been significantly increasing recently [8]. More specifically, the global consumption of REEs is predicted to expand at a 4.4% pace per year between 2016 and 2026 [9]. However, the significant levels of REEs being extracted and used in various industries have led to an increase in the REE discharge into the environment.

Once released into the environment, REEs can accumulate in the soil, water, plants, and in animals [10,11,12]. Eventually, this can impact human health by raising the concentrations of REEs within the human body. It has been shown that chronic exposure to rare earth elements can negatively impact the immune system, neurological system, digestive tract, circulatory system, and respiratory system. Furthermore, persistent exposure to REEs in youngsters may result in a drop in IQ [13]. In addition, studies have shown that chromosomal aberrations are among the anomalies caused by REEs [14].

Moreover, REE deposits are known as radioactive materials that occur naturally, containing a significant amount of uranium (^238^U) and thorium (^232^Th) radionuclides [15,16,17]. Activity concentrations of uranium and thorium in REEs-bearing minerals may reach up to 50,000 Bq/kg and 350,000 Bq/kg, respectively [15,18]. Extraction of REEs from these minerals may expose workers to gamma radiation and dust inhalation, which eventually might have a negative impact on people’s health [18]. Pneumoconiosis and pulmonary fibrosis are the most common diseases associated with inhaling REE dust [19,20]. However, it has not yet been determined whether these diseases are caused only by dust inhalation or whether they also occur as a result of radiological exposure [19]. Therefore, investigating the possible health effects of REEs on humans is essential.

The content of REEs in nature is shaped by various factors, including the type of parent rocks they originate from (e.g., bastnaesite, monazite, allanite), the processes of their weathering, and soil formation [21,22]. However, one of the major anthropogenic sources of REEs is coal combustion ash, also known as coal fly ash (CFA) [23].

CFA has long been seen as a possible source of REEs, and numerous studies have been conducted regarding to the determination of the REE content in CFA. Pan et al. [24] studied the REE content in various CFAs from a thermal power plant (TPP) in southern China. Their conclusions pointed out that CFA is a significant source of REEs, where the REEs content can range up to 530 mg/kg. Blissett et al. [25] research on the United Kingdom’s and Poland’s CFAs has revealed that CFA could potentially contain substantial REE reserves, with quantities reaching up to 480 mg/kg. Furthermore, Hower et al. [26] investigated the content and distribution of REEs in CFA from twenty-two TPPs in the United States and pointed out that the content of REEs reaches over 800 mg/kg, with gadolinium and thulium being the most abundant elements.

Although CFA is an important source of REEs, problems arise in the disposal of CFA since most countries in the world do not consider it as hazardous waste, and regulations for disposal are limited [27]. Therefore, if CFA landfills are not properly managed, the leaching of REEs from ash into the groundwater can occur [28,29,30]. As a result, REEs can contaminate groundwater, making it a significant issue for people, particularly for those who depend on groundwater for drinking. Groundwater pollution is a serious problem, as groundwater is one of the main sources of drinking water. In addition, it is used in agriculture and in various industrial processes [31]. Therefore, the supply of clean groundwater is a key factor for the safety and quality of life of every individual. It has also been noted that there appears to be a significant lack of studies concerning the REE content in groundwater and the risk that those elements pose to people’s health [32].

Hence, the aim of this work was to examine the content and distribution of REEs in groundwater in the vicinity of a large coal-fired power plant. In addition, the spatial distribution of REEs was investigated in order to determine potential hotspots. Pearson correlation analysis and principal component analysis were used to determine the relationship between REEs, as well as their potential source. Also, the health risk from REEs in groundwater was assessed regarding different population groups. This study thoroughly investigates the concentration of REEs in groundwater surrounding a coal-fired TPP. According to the authors’ knowledge, no prior studies have comprehensively analyzed the impact of such power plants on REE contamination in groundwater. The findings of this study provide essential insights into the behavior of REEs in groundwater and their potential risks to human health. Therefore, this work contributes to expanding the knowledge of the occurrence of REEs in groundwater, with a focus on coal-fired TPPs. It can also serve as an important parameter when adopting regulations related to groundwater quality in the investigated area since groundwater is one of the main drinking water sources in the area under study.

## 2. Materials and Methods

### 2.1. Study Area

The coal-fired TPP Nikola Tesla, also known as TENT, is situated in Serbia, in Southeast Europe. It belongs to the Obrenovac municipality, located within the Metropolis of Belgrade, the capital of Serbia. The municipality of Obrenovac covers an area of 411 km^2^, with an elevation of approximately 76 m. As to the 2011 census, Obrenovac had 72,524 residents, whilst the urban area had 25,429 residents. With an annual production of over 8000 GWh, TPP Nikola Tesla is the largest power plant in Serbia and one of the largest in Southeastern Europe. It is situated on the right bank of the Sava River. TPP Nikola Tesla A and TPP Nikola Tesla B are the two plants that make up the complex. Six generating units at TPP Nikola Tesla A have a capacity of 1650.5 MW, while two generation units at TPP Nikola Tesla B, which is situated 17 km upstream of TPP Nikola Tesla A, have a capacity of 1240 MW [33,34].

**Figure 1:**
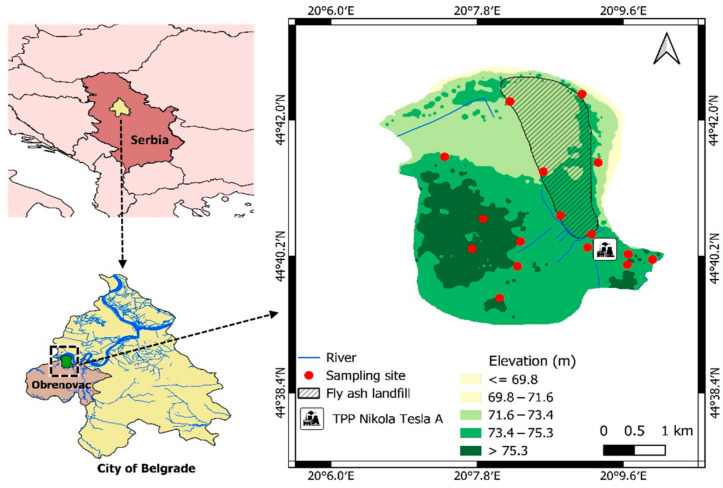
Study area with the locations of sampling points.

This study investigated the presence of rare earth elements (REEs) in the groundwater around TPP Nikola Tesla A. The study area, including an elevation profile and the location of the sampling points, is depicted in Figure 1. The power plant uses roughly 2.5 t/h of lignite coal, producing and disposing of approximately 2.4 Mt of ash annually. Additionally, a 900 ha surface area covered by ash landfills [35] is situated within the study area.

### 2.2. Sampling and Laboratory Analysis

A total number of 16 groundwater samples were collected from monitoring wells. Polyethylene bottles of 0.5 L volume were used as sampling containers, and each sample was collected in triplicates. Bottles were first washed with deionized water and then rinsed two to three times with the groundwater to be collected. Groundwater was sampled after it had been pumped for fifteen minutes. Each bottle was preserved by adding 2 mL of concentrated nitric acid, carefully labeled, stored at 4 °C, and transferred to the laboratory within a day.

The REE content in samples was determined using a Thermo Scientific ICP-MS instrument, model iCap Q (Waltham, MA, USA). The instrument was regularly calibrated using calibration standards made by diluting certified stock solution (PE-MECAL2-ASL-1 multi-element standard solution (10 mg/L each REE) from Accustandard Inc. (New Haven, CT, USA)). The ICP-MS instrument employed a plasma power of 1500 W. The instrument utilized an argon gas flow of 14 L/min, 0.85 L/min, and 0.96 L/min for cooling, auxiliary, and nebulization, respectively, along with a 5 mL/min helium gas flow in KED mode. This method enabled the correction of polyatomic interferences, including oxides and doubly charged ions. Samples were injected using a quartz injector with an internal diameter of 2.5 mm and nickel interface cones. The data acquisition process involved 20 sweeps per reading, with 3 replicates, 3 points per peak, and dwell times varying from 10 to 40 ms. The instrument ran in peak hopping scan mode. The sample flush, read delay, and wash times were set at 5, 25, and 55 s, respectively.

The quantification process used matrix-matched external calibration. Six standards that span the anticipated range of concentrations in the samples were used to create the calibration curves. For every REE, the linearity was confirmed by the high correlation coefficient (r^2^) value. Based on the calibration curve slope and the standard deviation of blank, the limit of detection (LOD) was calculated for each REE individually. The lowest concentration of each REE for each groundwater sample was more than three times higher than the LOD value. The precision of the method was evaluated by examining replicate samples comprising field and laboratory triplicates. The results showed that the precision of the method was within the acceptable range of ±5% of the relative standard deviation (RSD) between replicates. In order to determine recovery for each REE, a spiked water sample was utilized for every four samples. All REEs had average recoveries between 95% and 104%, while the expanded standard uncertainty (U) ranged from 5.2% to 11.5%. All validation parameters, including the calibration equations, R^2^, LOD, recovery, U, and RSD, are presented in Table S1 from the Supplementary Materials.

Considered the REE isotopes (along with their interferences) were: ^45^Sc (COO, COOH), ^89^Y, ^139^La, ^140^Ce, ^141^Pr, ^146^Nd, ^147^Sm, ^153^Eu (BaO), ^157^Gd (CeOH, PrO), ^159^Tb (NdO), ^163^Dy (SmO), ^165^Ho (SmO), ^166^Er (SmO, NdO), ^169^Tm (SmO, EuO), ^172^Yb (GdO), and ^175^Lu (GdO, TbO).

### 2.3. REE Distribution Pattern and Anomalies Calculation

To assess the REE distribution, normalization to the upper continental crust (UCC) [36] was carried out. This allows for the identification of anomalies or deviations from the standard. While normalizations to the Post-Archean Australian Shale (PAAS) and North American Shell Composites (NASC) can also be carried out, the distributional variations with respect to UCC were negligible and hence not relevant to the current investigation.

Depletion or enrichment of an element in relation to its neighboring REE is considered a negative or positive REE anomaly. In this study, Ce, Eu, Tm, and Tb anomalies were calculated using Equations (1)–(4) adapted from the previous literature [4,37,38] as follows:

(1) $$Ce/{Ce}^{*}=2{Ce}_{N}/({La}_{N}+{Pr}_{N})$$

(2) $$Eu/{Eu}^{*}=2{Eu}_{N}/({Sm}_{N}+{Gd}_{N})$$

(3) $$Tm/{Tm}^{*}=2{Tm}_{N}/({Er}_{N}+{Yb}_{N})$$

(4) $$Tb/{Tb}^{*}=2Tb/({Gd}_{N}+{Dy}_{N})$$

where *Ce*/Ce*, *Eu*/Eu*, *Tm*/Tm*, and *Tb*/Tb* represent Ce, Eu, Tm, and Tb anomalies, respectively, while *N* stands for the UCC-normalized concentration values of the element.

### 2.4. Health Risk Assessment

Non-carcinogenic and carcinogenic health risks for adults and children were estimated through hazard index (HI) and incremental lifetime cancer risk (ILCR) using the following equations [39,40]. Two exposure routes to contaminants were considered: ingestion and oral exposure. Calculation of HI and ILCR involves three steps.

In the first step, chronic daily intake (*CDI*) for both ingestion and oral exposure is calculated as follows:

(5) $${CDI}_{\;ing}^{\;}=\frac{C\times IR\times EF\times ED}{BW\times AT}$$

(6) $${CDI}_{\;derm}^{\;}=\frac{C\times SA\times Kp\times ET\times EF\times ED\times CF}{BW\times AT}$$

where C denotes the concentration of the detected pollutant in the groundwater of the study area, measured in mg/L; IngR signifies the rate of ingestion, which is 2.5 L/day for adults and 0.78 L/day for children; EF stands for the frequency of exposure, which is 365 days/year for ingestion and 350 days/year for dermal exposure, applicable to both adults and children; ED refers to the duration of exposure, which is 30 years for adults and 6 years for children; BW represents the average human body weight, and is 70 kg for adults and 15 kg for children; AT is the averaging time, which is 25,550 days for adults and 2190 days for children for non-carcinogenic risk, and 25,550 days for both adults and children for carcinogenic risk; SA is the area of exposure, which is 18,000 cm^2^ for adults and 6600 cm^2^ for children; ET stands for the time of exposure, which is 0.58 h/day for adults and 1 h/day for children; Kp is the coefficient of dermal permeability, and is 0.001 cm/h for all REEs; and CF is a unit conversion factor, which is 0.001 L/cm^3^ for both adults and children [39,41].

In the second step, the hazard quotient (HQ) is calculated as the ratio of chronic daily intake and reference dose (RfD) for both exposure routes, as follows.

(7) $${HQ}_{\;ing}^{\;}=\frac{{CDI}_{\;ing}^{\;}}{{RfD}_{ing}}$$

(8) $${HQ}_{\;derm}^{\;}=\frac{{CDI}_{\;derm}^{\;}}{{RfD}_{derm}}$$

In this study, a uniform reference dose (RfD) for all REEs was used, which was set at 0.02 mg/(kg × day) [1,14].

The last step involves the calculation of the hazard index, as presented in Equation (9).

(9) $$HI=\sum {(HQ}_{ing}+{HQ}_{derm})$$

HI values greater than 1 indicate significant non-carcinogenic health risk, while those lower than 1 suggest negligible non-carcinogenic risk [42,43].

After CDI is calculated, to determine carcinogenic risk, the carcinogenic risk index (CR) for both exposure routes is evaluated as follows:

(10) $${CR}_{ing}={CDI}_{ing}\times {SF}_{ing}$$

(11) $${CR}_{derm}={CDI}_{derm}\times {SF}_{derm}$$

where SF is the cancer slope factor set to 3.2 × 10^−12^ for all REEs [1,44].

In the ultimate step, ILCR is evaluated as follows:

(12) $$ILCR=\sum \left({CR}_{ingestion}+{CR}_{dermal}\right)$$

If the ILCR values are less than 1.0 × 10^−6^, the population exposed is at a negligible risk of developing cancer. However, if the ILCR exceeds 1.0 × 10^−4^, the risk of cancer is considered significant [45,46].

### 2.5. Data Analysis

To ensure that data follows normal distribution, a log transformation was applied to the data. Afterwards, the Ryan-Joiner test was used to confirm the normality of the transformed data. SPSS software version 23 was used for statistical analyses, including descriptive statistics, Pearson correlation analysis, and principal component analysis (PCA). Pearson correlation analysis and principal component analysis were performed to determine the linkage between REEs and similarity in their behavior, as previously thoroughly detailed elsewhere [47,48]. Statistical analyses were considered significant if *p* < 0.05. Spatial variation maps were conducted in QGIS software version 3.30 using the inverse distance weighting method, while other graphs were carried out in R software packages version 4.1.2.

## 3. Results and Discussion

### 3.1. Occurrence and Geospatial Variation of REEs in Groundwater

**Figure 2:**
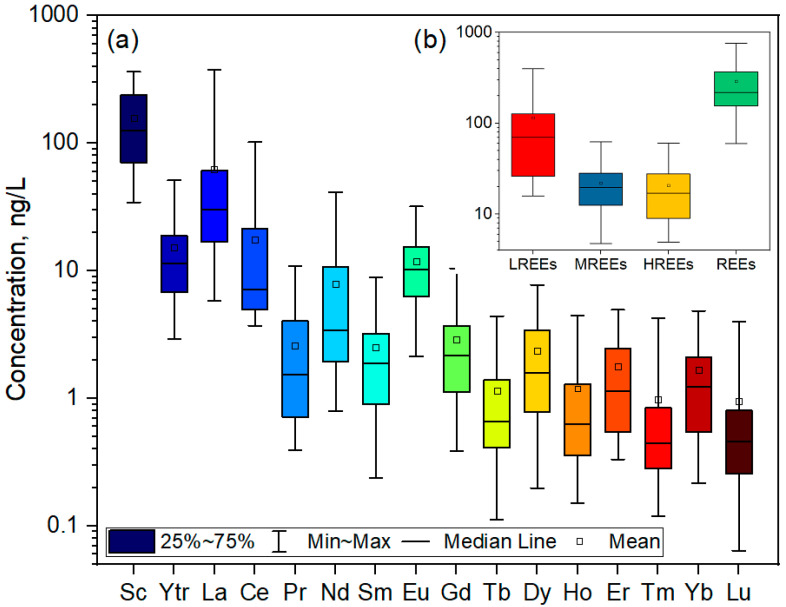
Box plot for 16 different REEs (**a**), LREEs, MREEs, HREEs, and REEs (**b**) concentration in groundwater.

Descriptive statistics for 16 analyzed REEs are presented in Table S2 from the Supplementary Materials and depicted in box plots in Figure 2. The total REE concentration in the groundwater ranged from 59.9 ng/L to 758 ng/L, with an average value of 290 ng/L. The total LREE concentration was significantly higher than that of MREEs and HREEs. Additionally, the concentration of LREEs varied between 15.8 ng/L and 397 ng/L, while the concentration of MREEs ranged between 4.3 ng/L and 60.7 ng/L, with an average of 114 ng/L and 20.8 ng/L, respectively. The total HREE concentration ranged between 5.3 ng/L and 61.8 ng/L, with an average value of 21.8 ng/L.

A notable REE fractionation was found in the examined groundwater, with all samples showing the ratio of LREEs/HREEs greater than 1. These results showed that LREEs comprise around 78.9% of the total REE concentration. Similar findings regarding the LREE/MREE ratio were observed, indicating LREE enrichment in the investigated area. More specifically, the LREE/HREE ratios ranged from 1.1 to 47.2, while LREEs/MREEs ratios varied between 1.0 and 47.0, showing significant enrichment in LREEs compared to HREEs and MREEs.

**Figure 3:**
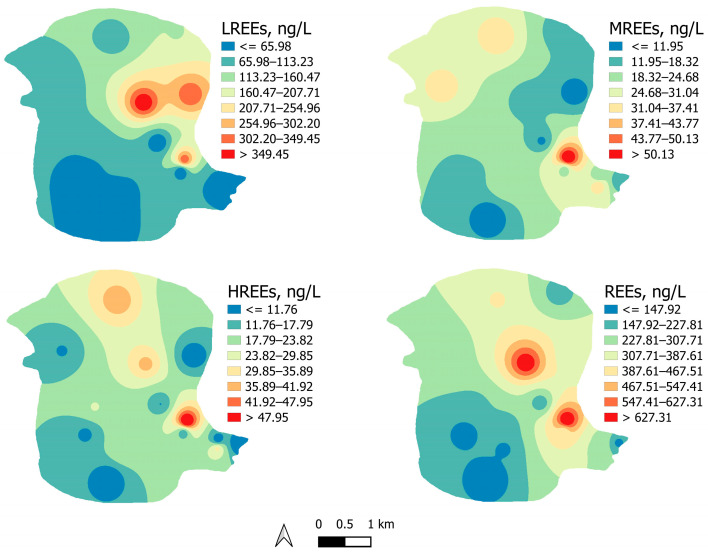
Spatial variation of LREEs, MREEs, HREEs, and REEs in the investigated area.

Spatial variation of REEs, LREEs, MREEs, and HREEs is presented in Figure 3. The highest concentrations were observed near the TPP and ash landfill, decreasing with the distance from the plant and the landfill. The average concentration of REEs followed the order: Sc (157 ng/L) > La (62.2 ng/L) > Ce (17.5 ng/L) > Y (15.2 ng/L) > Eu (11.9 ng/L) > Nd (7.9 ng/L) > Gd (2.9 ng/L) > Pr (2.6 ng/L) > Sm (2.5 ng/L) > Dy (2.3 ng/L) > Er (1.8 ng/L) > Yb (1.7 ng/L) > Ho (1.2 ng/L) > Tb (1.1 ng/L) > Tm (0.98 ng/L) > Lu (0.94 ng/L). The most abundant element was Sc, followed by La, accounting for 54.2% and 21.4% of the total REE concentration.

The average REE concentration in this study aligns with the reported concentrations of REEs in regions such as Jiangxi Province [49] and Simao Basin [50] in China (Table 1). However, some studies have reported higher and lower concentrations of REEs in groundwater. For instance, the concentrations of REEs in the Polish Lowlands in eastern Poland were found to be relatively high [51], while the concentrations in the Alpine aquifers in Switzerland [52] or Romagna area in southeastern Italy [53] were reported to be relatively low. Furthermore, very high concentrations of REEs were found within aquifers in Ogun state in Nigeria, reaching up to 232,000 ng/L [13]. Similarly, Imphal Valley in India exhibits an average REE concentration of 57,000 ng/L [54]. These variations in REE concentrations can be attributed to a multitude of factors, including the geochemical characteristics of the region, the type of rock minerals present, the environmental conditions, and anthropogenic effects [55,56]. Therefore, while our study contributes valuable data to the existing body of knowledge, it also underscores the need for continued research in this area to better understand the mechanisms that control the concentration and distribution of REEs in groundwater.

It would be beneficial to incorporate additional sampling points in the area in future studies to enhance the investigation of the distribution of REEs. Further research should also address radiological aspects by analyzing uranium and thorium concentrations.

### 3.2. REE Distribution Pattern and Anomalies

**Figure 4:**
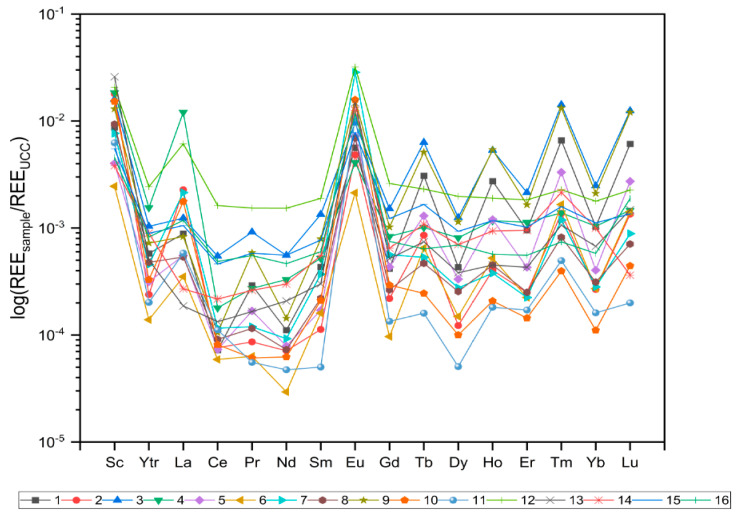
REEs distribution patterns normalized to the UCC.

REE distribution patterns were assessed by the UCC-normalized REE values, the ratios of (La/Yb)_N_, (La/Sm)_N_, and (Gd/Yb)_N_, and Ce, Eu, Tb, and Tm anomalies. Figure 4 shows UCC-normalized REE concentration patterns for the groundwater samples, while Table 2 summarizes values for the (La/Yb)_N_, (La/Sm)_N_, and (Gd/Yb)_N_ ratios and anomalies. As seen in Figure 4, most groundwater samples showed little HREE and MREE enrichment compared to LREEs. In addition, the ratios of (La/Yb)_N_, (La/Sm)_N_, and (Gd/Yb)_N_ can be used to represent the fractionation between LREEs and HREEs, LREEs and MREEs, and MREEs and HREEs, respectively, where N denotes for the UCC-standardized values of the elements’ concentration. The results showed that (La/Yb)_N_ ranged between 0.27 and 16.0, with an inclination to rise towards the coal-fired power plant. A similar pattern was observed concerning the (La/Sm)_N_ ratio, which varied from 0.49 to 23.19, and the (Gd/Yb)_N_ ratio, ranging from 0.36 to 2.65. Therefore, for the groundwater in the study area, REE levels rise from light to medium and heavy.

Furthermore, REE patterns were found to be non-smooth, exhibiting negative and positive anomalies for particular REEs. In addition, the results showed that Ce is depleted in comparison to the other REEs, with all samples showing negative Ce anomaly, ranging from 0.03 to 0.82, with an average value of 0.33. Positive Ce anomaly is typically associated with the enrichment in HREEs, whereas negative Ce anomaly suggests LREEs enrichment [68]. Furthermore, positive Ce anomaly implies the presence of highly soluble Ce(III), whereas negative Ce anomaly suggests Ce(III) oxidation to weakly soluble Ce(IV) and is usually associated with high alkalinity or high pH values [6,37]. Contrarily, Eu is enriched compared with other REEs. Major positive Eu anomaly was observed for all groundwater samples. The Eu/Eu* value varied between 6.05 and 117, with an average of 30.0. Positive Eu anomaly is widespread in waters and indicates a hydrogeochemical system that is yet to achieve an equilibrium state and the condition that represents rock dissolution [51,69].

The highest Eu and Ce anomalies were observed near the power plant, suggesting possible anthropogenic-related causes of these anomalies. Tb anomaly ranged between 0.88 and 7.27, with an average value of 2.76. Most of the samples (93.7%) showed positive Tb anomaly, while the rest exhibited negative Tb anomaly. Furthermore, all samples exhibited major positive Tm anomaly, ranging from 1.26 to 7.98, with an average value of 3.94.

Unlike the Ce and Eu anomalies, Tb and Tm anomalies had the highest values at the edges of the investigated area, further away from the TPP, where agricultural areas are situated. In addition, REEs, including Tb and Tm, are utilized as additions in fertilizers [55,70]. Over time, repeated application of these agricultural products can result in the accumulation of these elements in the groundwater.

### 3.3. Multivariate Statistics

**Figure 5:**
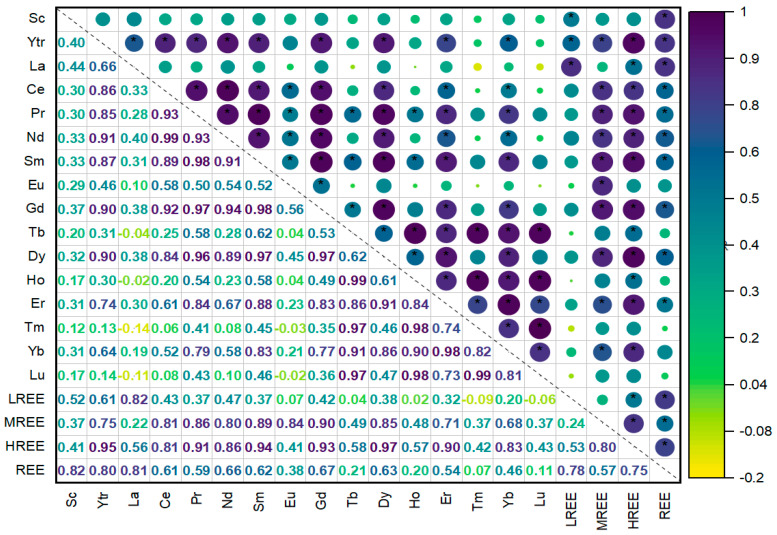
Pearson correlation analysis of REEs in groundwater around the Nikola Tesla power plant. Correlation coefficient value significant at the 0.05 level is marked with asterisk (*).

Pearson correlation analysis showed a high linkage between different REEs (Figure 5). Pearson correlation coefficient (r) ranged between −0.14 and 1.0. A positive r value indicates a positive correlation between variables. Conversely, a negative r value indicates a negative correlation. In addition, two variables exhibit a strong correlation if 0.7 < |*r*| < 1.0, moderate if 0.5 < |*r*| < 0.7, and weak if |*r*| < 0.5 [71]. The majority of REEs were positively correlated, except for Tb with La (r = −0.04), Ho with La (r = −0.02), Tm with La (r = −0.14), Tm with Eu (r = −0.03), Lu with La (r = −0.11), and Lu with Eu (r = −0.02). Furthermore, La and Eu did not show strong correlations with the other REEs, suggesting that they may behave differently from the rest of the REEs in the investigated area. Similar observations were concluded with regard to Sc.

Concerning La, the highest correlation was observed with Y (r = 0.66), while Eu correlated the most with Ce (r = 0.58). However, the rest of the REEs showed strong correlations with each other. Tb showed the strongest linkage with Lu, Tm, and Ho (r = 0.97, r = 0.97, and r = 0.99, respectively), while Sm exhibited the strongest correlation with Pr, Dy, and Gd (r = 0.98, r = 0.97, and r = 0.98, respectively). Additionally, Tm showed the strongest correlation with Ho (r = 0.98) and Lu (r = 0.99). Furthermore, there was a substantial correlation between particular elements and LREEs, MREEs, HREEs, and REEs. The REE significant correlations indicated that the REEs in the study area shared common geochemical characteristics and originated from similar sources. The comparable distribution patterns of REEs in groundwater (Figure 4) for all samples suggested that all REEs have had similar origins. REE behavior was further discussed using principal component analysis.

**Figure 6:**
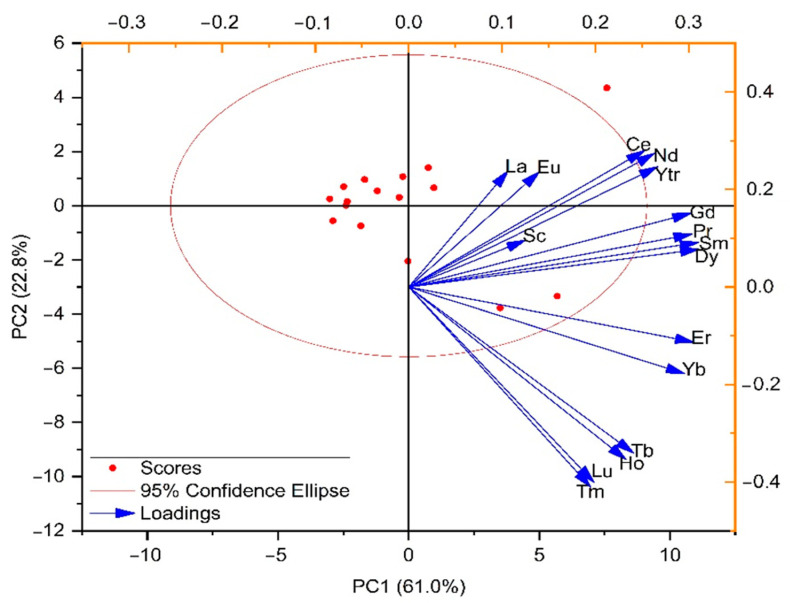
Principal component loading and score plot for the first two principal components of REEs in the groundwater around the Nikola Tesla power plant.

Principal component analysis extracted three components with eigenvalues greater than one, comprising 91.1% of the total variance (Table S3 from Supplementary Materials). The biplot for the first two components is presented in Figure 6. The first component had strong loadings of Ce, Y, Pr, Nd, Gd, Sm, and Dy, accounting for 61.0% of the total variance. The second component explained 22.8% of the total variance, with strong loadings of Tm, Tb, Ho, Er, Yb, and Lu. The first two components were distinguished by LREEs and HREEs, which indicated a clear separation between the two and was consistent with the LREEs/HREEs ratios. The third component, with 7.3% of the variance explained, showed strong positive loadings of Sc and La. The separation of Sc and La from the other REEs confirms their different behavior in groundwater in the investigated area.

### 3.4. Health Risk Assessment

Non-carcinogenic and carcinogenic health risks that REEs in groundwater pose to people around TPP Nikola Tesla are presented in Table S4 from the Supplementary Materials. Regarding non-carcinogenic risk, HI for adults (HIa) ranged from 1.07 × 10^−4^ to 1.36 × 10^−3^, while HI for children (HIc) ranged between 1.56 × 10^−4^ and 1.98 × 10^−3^. Children were approximately 1.5 times more at risk than adults, with the average HIc values of 7.56 × 10^−4^ and 5.19 × 10^−4^, respectively. The results showed that the ingestion exposure route contributed more to the non-carcinogenic risk than the dermal exposure route. Since groundwater ingestion is the main route of exposure to REEs, typical hand-to-mouth actions may provide a larger danger to children than adults [14]. In addition, children’s lower body weight also may be the reason for higher non-carcinogenic risk in children [72,73].

**Figure 7:**
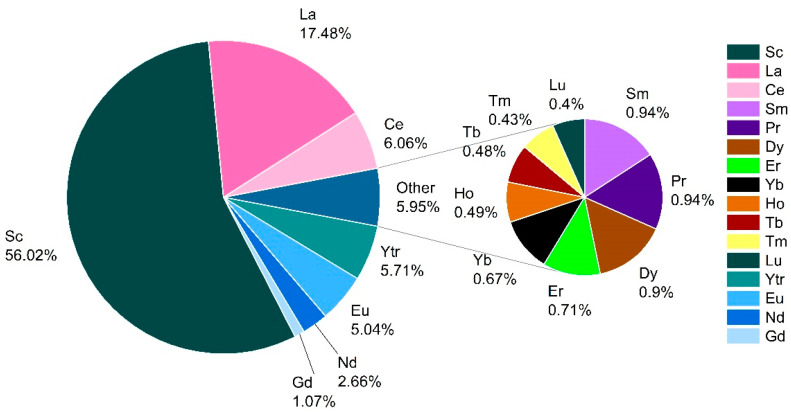
Contribution of different REEs to the overall non-carcinogenic health risk for adults.

Among all REEs, the highest average HQ values showed Sc (HQ = 4.09 × 10^−4^), followed by La (HQ = 1.63 × 10^−4^), while Lu had the lowest HQ values (HQ = 2.48 × 10^−6^), regarding both population groups. In addition, Sc was responsible for 56.0% of the non-carcinogenic risk in adults and 55.9% of the non-carcinogenic risk in children (Figure 7 and Figure S1 from the Supplementary Materials).

With regard to carcinogenic risk, ILCR for adults (ILCRa) ranged between 2.02 × 10^−18^ and 2.68 × 10^−17^, while ILCR for children (ILCRc) varied between 8.08 × 10^−18^ and 1.02 × 10^−17^. Unlike the non-carcinogenic risk, the carcinogenic risk for adults was approximately 2.5 higher compared to children, with average ILCRa values of 9.76 × 10^−18^ and 3.91 × 10^−18^, respectively, which also can be explained by children’s lower body weight [74]. Ingestion rate is found to be the main exposure route to carcinogenic risk from groundwater REEs. Furthermore, the contribution of the REEs to the ILCR was similar for the HI, with Sc accounting for 51.41% and 56.10% of the carcinogenic risk for adults and children, respectively (Figures S2 and S3 from the Supplementary Materials).

**Figure 8:**
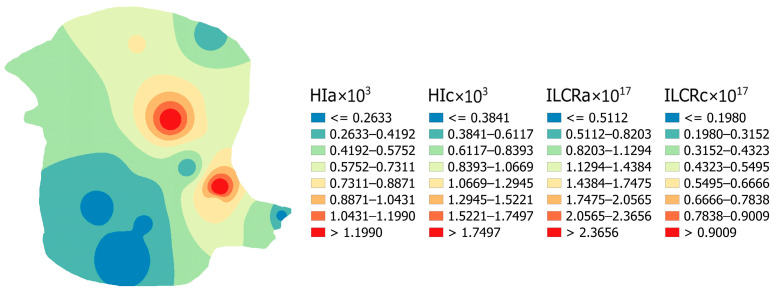
Spatial distribution of non-carcinogenic and carcinogenic health risk of REEs for both population groups.

The spatial variability of non-carcinogenic and carcinogenic health risks for both population groups in the investigated area is depicted in Figure 8. All risks showed the same distribution patterns. The highest risk is observed near the power plant and CFA landfills. The risk decreased with increasing distance from the power plant. However, the health risk assessment results showed that all of the samples had HI and ILCR values below the permissible limits of 1 and 1.0 × 10^−6^, respectively, suggesting that REEs are unlikely to pose significant health risks to humans in the area studied.

Nevertheless, the ongoing ash disposal in the area can lead to the release of REEs from ash landfills. Furthermore, as agriculture plays an important role in the area, REEs can accumulate in arable land and be absorbed by crops, endangering the health of the local population, even in locations remote from the power plant. Therefore, the amount of REEs in groundwater must be continuously monitored. In view of this, various techniques can be used for environmental remediation to reduce exposure and risks, including chemical precipitation, ion exchange, membrane filtration, and adsorption onto minerals, such as clay or different biomaterials [75,76]. Overall, environmental remediation can help lower environmental concentrations and human exposure to rare earth elements over time.

## 4. Conclusions

This study investigated the presence and spatial variability of rare earth elements (REEs) in groundwater surrounding a major coal-fired power plant in Serbia. All tested groundwater samples showed the presence of REEs, with concentrations ranging from 59.9 to 758 ng/L and an average of 290 ng/L. The LREEs were found to be the most abundant, constituting over 78% of the total REEs. The spatial variation of REEs exhibited higher concentrations in close proximity to the power plant and its associated ash landfill, gradually decreasing with distance. Furthermore, the study identified LREEs enrichment compared to MREEs and HREEs, with distinct fractionation patterns and anomalies observed. The findings from multivariate analyses, including Pearson correlation and principal component analyses, indicated strong correlations among most REEs, suggesting common geochemical characteristics and sources. The health risk assessment revealed HI and ILCR below permissible limits, indicating that the current levels of REEs in the groundwater do not pose significant health risks to the population in the investigated area. However, potential future risks associated with ongoing ash disposal and the accumulation of REEs in the environment emphasize the need for continuous monitoring to assess any long-term impacts on human health and the environment.

## Figures and Tables

**Table 1:** REE concentrations at different locations in the world compared to the present study.

Region	REE Conc. Range, ng/L	REE Mean Conc., ng/L	Reference
Polish Lowlands, Poland	0.6–10,103	559.3	[51]
Jiangxi Province, China	90.0–540.0	300.0	[49]
Simao Basin, China	58.0–783.0	220.0	[50]
North China Plain, China	81.2–163.3	109.0	[57]
Anhui Province, China	21.8–315.8	103.5	[58]
Alpine aquifers, Switzerland	2.60–67.00	23.90	[52]
Aspo Hard Rock Laboratory, Sweden	90.0–880	362.9	[59]
Sikhote Alin, Russia	40.0–920.0	450.0	[60]
Teviot Brook catchment, Australia	18.0–447	61.0	[61]
Romagna area, Italy	28.0–87.0	42.0	[53]
Abbruzo region, Italy	53.0–814	330.3	[62]
Mount Vulture Basin, Italy	19.8–947.1	203.2	[63]
Mizunami URL, Japan	9.3–119.1	48.7	[64]
Dindigul District, India	23.0–16,000	849.0	[65]
Imphal Valley, India	54,000–63,000	57,000	[54]
Majuli Island, India	1270–5710	3094	[66]
Bam Plain, Iran	180.0–8360	1250	[67]
Ogun State, Nigeria	1140–232,000	22,600	[13]
Obrenovac, Serbia	59.9–758	290	this study

**Table 2:** REEs anomalies and fractionation normalized to the UCC.

Sample	Eu/Eu*	Ce/Ce*	Tm/Tm*	Tb/Tb*	(La/Sm)_N_	(La/Yb)_N_	(Gd/Yb)_N_
1	13.3	0.12	6.65	7.27	2.05	0.86	0.41
2	29.1	0.06	5.31	5.02	20.2	8.54	0.82
3	6.70	0.51	6.14	4.55	0.92	0.50	0.61
4	6.05	0.03	1.28	1.23	23.2	11.6	0.80
5	23.8	0.20	7.98	3.40	3.25	1.37	1.07
6	16.6	0.29	6.69	5.28	2.18	1.29	0.36
7	61.1	0.10	4.69	1.27	5.78	7.60	2.00
8	28.7	0.28	2.91	1.80	2.43	1.72	0.85
9	17.5	0.15	7.04	4.73	1.05	0.39	0.48
10	62.8	0.09	3.11	1.25	8.42	16.0	2.65
11	117	0.35	2.99	1.73	11.6	3.62	0.83
12	14.2	0.42	1.26	1.01	3.24	3.44	1.46
13	36.3	0.76	1.93	1.66	0.62	0.28	0.77
14	21.0	0.82	2.21	1.61	0.49	0.27	0.65
15	7.82	0.56	1.49	1.54	1.37	0.94	1.10
16	17.5	0.56	1.31	0.88	1.96	2.04	1.30
Min	6.05	0.03	1.26	0.88	0.49	0.27	0.36
Max	118	0.82	7.98	7.27	23.2	16.0	2.65
Average	30.0	0.33	3.94	2.76	5.55	3.78	1.01

## Data Availability

The data presented in this study are available on request from the corresponding author.

## Supplementary Materials

The following supporting information can be downloaded at: https://www.mdpi.com/article/10.3390/toxics12010062/s1, Table S1. Validation parameters of REEs in groundwater samples using ICP-MS, including the calibration equations (A·x + B), calibration correlation coefficient (r2), the limit of detection (LOD), recovery (R), relative standard deviation (RSD), and expanded standard uncertainty (U); Table S2. Descriptive statistics of REEs in groundwater (ng/L); Table S3. Principal component analysis results showing three extracted components with eigenvalues greater than one and their loadings; Table S4. The results of human health risk assessment of REEs in groundwater; Figure S1. Contribution of 16 analyzed REEs to the overall non-carcinogenic health risk for children; Figure S2. Contribution of 16 analyzed REEs to the overall carcinogenic health risk for adults; Figure S3. Contribution of 16 analyzed REEs to the overall carcinogenic health risk for children.
