# Supplementary material for: Spatial Variability of Rare Earth Elements in Groundwater in the Vicinity of a Coal-Fired Power Plant and Associated Health Risk
Source: Toxics. 2024 Jan 12;12(1):62. doi: 10.3390/toxics12010062 (PMC10820410; doi:10.3390/toxics12010062)
Supplement: Supplementary file 1 [file toxics-12-00062-s001.zip › toxics-2795381-supplementary.pdf]

Supplementary Materials

Spatial Variability of Rare Earth Elements in Groundwater in the Vicinity of a Coal-Fired Power Plant and Associated Health Risk

Jelena Vesković [1], Milica Lučić [2], Mirjana Ristić [1], Aleksandra Perić-Grujić [1], Antonije Onjia [1,*]

[1]*University of Belgrade, Faculty of Technology and Metallurgy, Karnegijeva 4, 11120 Belgrade, Serbia*
[2]*Innovation Center of the Faculty of Technology and Metallurgy, Karnegijeva 4, 11120 Belgrade, Serbia*
[*] *Correspondence: onjia@tmf.bg.ac.rs*

Table S1. Validation parameters of REEs in groundwater samples using ICP-MS, including the calibration equations ($A{\cdot}x{+}B$), calibration correlation coefficient (r^2), the limit of detection (LOD), recovery (R), relative standard deviation (RSD), and expanded standard uncertainty (U)

Element	A·x+B	r^2	LOD ng/L	R (%)	RSD (%)	U^* (%)
^{45}Sc	18.63·x + 6.667	0.9989	2.35	96	4.9	11.5
^{89}Y	161.4·x + 7.001	0.9994	0.852	98	1.6	5.8
^{139}La	409.9·x + 62.35	0.9984	0.257	95	2.1	6.4
^{140}Ce	404.0·x + 23.34	0.9999	0.187	101	2.4	7.1
^{141}Pr	439.1·x + 33.35	0.9997	0.079	96	1.5	5.6
^{146}Nd	87.67·x + 6.002	0.9999	0.123	97	3.4	8.7
^{147}Sm	76.32·x + 5.123	0.9997	0.031	96	1.6	5.7
^{153}Eu	292.9·x + 16.42	0.9999	0.059	101	1.4	5.7
^{157}Gd	109.2·x + 6.667	0.9967	0.016	95	1.3	5.3
^{159}Tb	553.4·x + 42.10	0.9975	0.022	104	2.1	6.8
^{163}Dy	151.4·x + 11.29	0.9998	0.014	102	1.1	5.4
^{165}Ho	552.6·x + 43.32	0.9997	0.031	97	2.2	6.6
^{166}Er	209.9·x + 17.47	0.9997	0.082	99	1.3	5.5
^{169}Tm	589.5·x + 38.18	0.9970	0.026	104	3.4	8.9
^{172}Yb	150.7·x + 12.26	0.9994	0.045	103	0.7	5.2
^{175}Lu	442.7·x + 34.29	0.9985	0.011	96	1.5	5.6

* – *Calculated from the RSD uncertainty and the uncertainty of spiked sample recovery* ($u = \sqrt{u(RSD)^2 + u(R)^2}$) *and the coverage factor 2.*

Table S2. Descriptive statistics of REEs in groundwater (ng/L)

Variable	Mean±U	Median±U	Minimum±U	Maximum±U	SD	S	K
Sc	157±18.0	126±14.5	34.4±3.93	363±41.7	99.3	0.59	-0.71
La	15.2±0.91	30.1±1.7	5.81±0.338	375±21.8	94.4	2.88	8.67
Ce	62.2±4.02	7.18±0.46	3.73±0.239	102±6.54	24.8	3.01	10.01
Pr	17.5±1.20	1.53±0.11	0.39±0.028	10.9±0.77	2.84	1.97	4.21
Nd	2.59±0.15	3.44±0.19	0.79±0.044	41.4±2.33	10.2	2.64	8.12
Sm	7.86±0.68	1.89±0.16	0.24±0.021	8.91±0.77	2.31	1.76	3.23
Eu	2.50±0.14	10.2±0.59	2.13±0.122	32.0±1.84	8.31	1.40	1.68
Gd	11.9±0.68	2.16±0.12	0.39±0.022	10.4±0.59	2.57	1.89	4.27
Tb	2.89±0.15	0.65±0.03	0.11±0.006	4.42±0.24	1.24	1.83	2.72
Dy	1.15±0.08	1.58±0.11	0.20±0.014	7.73±0.52	2.05	1.31	1.73
Ho	2.34±0.13	0.63±0.03	0.15±0.008	4.48±0.24	1.39	1.85	2.49
Er	1.19±0.08	1.14±0.08	0.33±0.022	4.94±0.33	1.48	0.96	-0.18
Tm	1.78±0.10	0.45±0.02	0.12±0.007	4.26±0.24	1.30	2.06	3.18
Yb	0.99±0.08	1.23±0.11	0.22±0.020	4.86±0.43	1.42	1.11	0.36
Lu	1.67±0.09	0.46±0.02	0.06±0.003	3.99±0.21	1.25	2.02	3.00
Y	0.94±0.05	11.3±0.64	2.92±0.164	51.1±2.87	12.3	1.88	4.17
LREEs	114	69.6	15.8	397	129	1.50	0.80
MREEs	20.8	19.4	4.31	60.7	13.7	1.71	3.94
HREEs	21.8	17.5	5.32	61.8	16.2	1.17	0.96
REEs	290	217	59.9	758	201	1.40	1.49

SD - standard deviation; S – skewness; K – kurtosis; U – uncertainty.

Table S3. Principal component analysis results showing three extracted components with eigenvalues greater than one, and their loadings

Variable	PC1	PC2	PC3
Sc	0.125	0.096	0.572
Y	0.267	0.247	0.159
La	0.106	0.238	0.669
Ce	0.253	0.280	-0.197
Pr	0.304	0.109	-0.160
Nd	0.264	0.274	-0.123
Sm	0.311	0.092	-0.112
Eu	0.140	0.236	-0.298
Gd	0.303	0.151	-0.081
Tb	0.241	-0.340	0.009
Dy	0.311	0.078	-0.039
Ho	0.233	-0.354	0.035
Er	0.306	-0.113	0.079
Tm	0.195	-0.410	0.009
Yb	0.296	-0.178	0.052
Lu	0.198	-0.400	0.035
Eigenvalue	9.763	3.649	1.172
Percentage of variance	61.02	22.80	7.33
Cumulative	61.02	83.83	91.15

Table S4. The results of human health risk assessment of REEs in groundwater

Health risk	REE	Population group					
		Children			Adults		
		Mean	Max	Min	Mean	Max	Min
HQ	Sc	4.09E-04	9.44E-04	8.95E-05	2.81E-04	6.48E-04	6.15E-05
	Y	3.96E-05	1.33E-04	7.59E-06	2.72E-05	9.13E-05	5.21E-06
	La	1.63E-04	9.82E-04	1.52E-05	1.11E-04	6.72E-04	1.04E-05
	Ce	4.58E-05	2.68E-04	9.76E-06	3.13E-05	1.83E-04	6.68E-06
	Pr	6.79E-06	2.86E-05	1.03E-06	4.65E-06	1.96E-05	7.06E-07
	Nd	2.06E-05	1.08E-04	2.08E-06	1.41E-05	7.42E-05	1.42E-06
	Sm	6.55E-06	2.33E-05	6.19E-07	4.48E-06	1.60E-05	4.23E-07
	Eu	3.12E-05	8.38E-05	5.60E-06	2.14E-05	5.73E-05	3.83E-06
	Gd	7.57E-06	2.74E-05	1.01E-06	5.18E-06	1.87E-05	6.94E-07
	Tb	3.00E-06	1.16E-05	2.94E-07	2.05E-06	7.92E-06	2.01E-07
	Dy	6.14E-06	2.03E-05	5.19E-07	4.20E-06	1.39E-05	3.55E-07
	Ho	3.13E-06	1.17E-05	3.96E-07	2.14E-06	8.03E-06	2.71E-07
	Er	4.66E-06	1.30E-05	8.70E-07	3.19E-06	8.86E-06	5.95E-07
	Tm	2.58E-06	1.12E-05	3.12E-07	1.77E-06	7.64E-06	2.13E-07
	Yb	4.37E-06	1.27E-05	5.69E-07	2.99E-06	8.72E-06	3.89E-07
	Lu	2.48E-06	1.05E-05	1.68E-07	1.69E-06	7.16E-06	1.15E-07
HI	Total	7.56E-04	1.98E-03	1.56E-04	5.19E-04	1.36E-03	1.07E-04
CR	Sc	2.12E-18	4.89E-18	4.64E-19	4.83E-18	1.12E-17	1.06E-18
	Y	2.05E-19	6.89E-19	3.93E-20	4.68E-19	1.57E-18	8.97E-20
	La	8.38E-19	5.05E-18	7.83E-20	1.91E-18	1.15E-17	1.79E-19
	Ce	2.35E-19	1.38E-18	5.02E-20	8.05E-19	4.72E-18	1.72E-19
	Pr	3.49E-20	1.47E-19	5.31E-21	1.19E-19	5.03E-19	1.82E-20
	Nd	1.06E-19	5.58E-19	1.07E-20	3.62E-19	1.91E-18	3.66E-20
	Sm	3.37E-20	1.2E-19	3.18E-21	1.15E-19	4.11E-19	1.09E-20
	Eu	1.61E-19	4.31E-19	2.88E-20	5.49E-19	1.47E-18	9.84E-20
	Gd	3.89E-20	1.41E-19	5.22E-21	1.33E-19	4.81E-19	1.78E-20
	Tb	1.54E-20	5.96E-20	1.51E-21	5.28E-20	2.04E-19	5.16E-21
	Dy	3.16E-20	1.04E-19	2.67E-21	1.08E-19	3.56E-19	9.13E-21
	Ho	1.61E-20	6.04E-20	2.04E-21	5.5E-20	2.06E-19	6.96E-21
	Er	2.4E-20	6.66E-20	4.48E-21	8.2E-20	2.28E-19	1.53E-20
	Tm	1.33E-20	5.75E-20	1.6E-21	4.54E-20	1.97E-19	5.49E-21
	Yb	2.25E-20	6.55E-20	2.93E-21	7.69E-20	2.24E-19	1.00E-20
	Lu	1.27E-20	5.38E-20	8.63E-22	4.35E-20	1.84E-19	2.95E-21
ILCR	Total	3.91E-18	1.02E-17	8.08E-19	9.76E-18	2.68E-17	2.02E-18

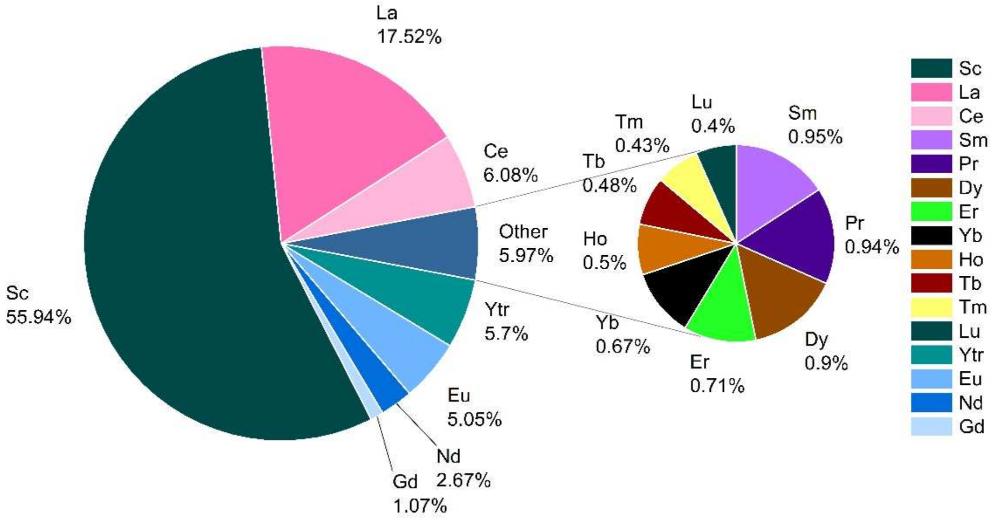

Figure S1. Contribution of 16 analyzed REEs to the overall non-carcinogenic health risk for children

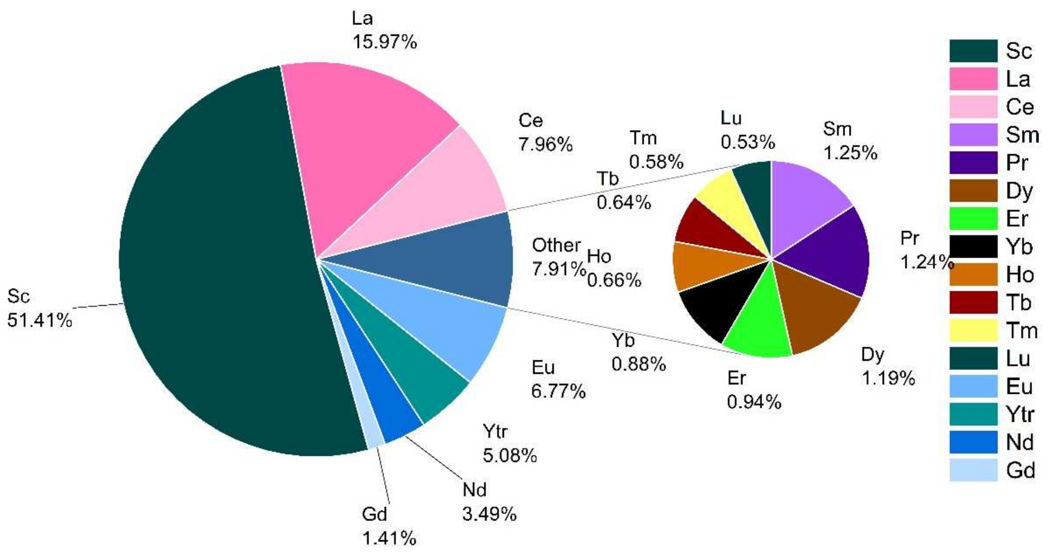

Figure S2. Contribution of 16 analyzed REEs to the overall carcinogenic health risk for adults

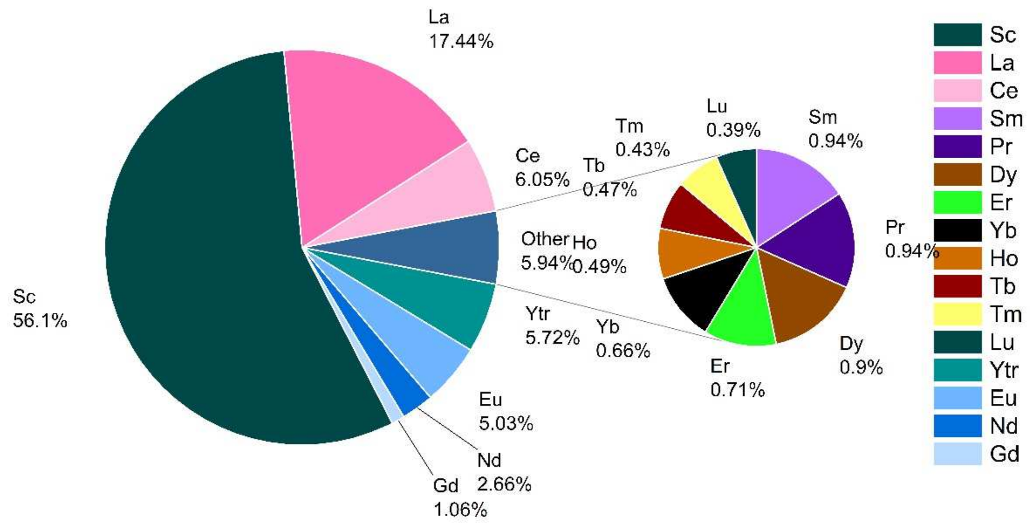

Figure S3. Contribution of 16 analyzed REEs to the overall carcinogenic health risk for children
